# Evaluating a Nationwide Neonatal Research Infrastructure: A Bibliometric Network Analysis of the Korean Neonatal Network

**DOI:** 10.3390/healthcare14142188

**Published:** 2026-07-20

**Authors:** Seong Wan Kim, Yujin Kwon, Yoong-A Suh, Jang Hoon Lee, Yun Sil Chang, Moon Sung Park, Seoheui Choi

**Affiliations:** 1 Department of Pediatrics, Ajou University School of Medicine, 164 World Cup-ro, Yeongtong-gu, Suwon 16499, Republic of Korea; kswan92@aumc.ac.kr (S.W.K.); mary5531@aumc.ac.kr (Y.-A.S.); neopedlee@aumc.ac.kr (J.H.L.); mspark@aumc.ac.kr (M.S.P.); 2 Medical Information & Media Center, Ajou University School of Medicine, 164 World Cup-ro, Yeongtong-gu, Suwon 16499, Republic of Korea; yujinkwon@aumc.ac.kr; 3 Department of Pediatrics, Samsung Medical Center, Sungkyunkwan University School of Medicine, 81 Irwon-ro, Gangnam-gu, Seoul 06351, Republic of Korea; yunsil.chang@gmail.com

**Keywords:** very low birth weight infant, extremely low birth weight infant, factual databases, bibliometrics, data visualization, bibliography of medicine, bibliographic databases

## Abstract

**Highlights:**

**What are the main findings?**

- Research based on the Korean Neonatal Network (KNN) has rapidly expanded over the past decade and has followed major global research trends in very low birth weight infants (VLBWIs).
- Research topics have evolved from observational and epidemiological studies toward analyses of neurodevelopmental outcomes, risk factors, and data-driven approaches including machine learning.

**What are the implications of the main findings?**

- National neonatal registries such as the KNN play an increasingly important role in generating evidence and supporting collaborative neonatal research.
- Identifying research trends and emerging topics may help guide future research priorities and optimize the use of large-scale clinical databases in neonatal medicine.

**Abstract:**

**Background/Objectives**: Since the establishment of the Korean Neonatal Network (KNN) in 2013, numerous studies have been conducted using its nationwide registry of very low birth weight infants (VLBWIs). Beyond serving as a clinical database, the KNN has evolved into a national healthcare research infrastructure supporting multicenter collaboration, evidence generation, and quality improvement in neonatal care. This study aimed to evaluate the scientific productivity, collaborative structure, and knowledge-generation capacity of the KNN through bibliometric network analysis and to identify future research directions in Korean neonatal research. **Methods**: Publications from 1 January 2015 to 5 June 2024 were retrieved from the Web of Science Core Collection. Literature searches were performed using Medical Subject Headings (MeSH), Emtree terms, and related keywords associated with very low birth weight infants, extremely low birth weight infants, and the Korean Neonatal Network. A total of 7060 global publications and 86 KNN-related publications were identified for analysis. Co-authorship and keyword co-occurrence analyses were conducted using VOSviewer to evaluate international collaborations, research themes, and temporal trends in keyword emergence. Scimago Graphica was used to visualize keyword evolution, and burst detection analysis was performed using CiteSpace to identify emerging research topics. **Results**: Among global publications on VLBWIs, South Korea ranked 13th worldwide with 226 publications and demonstrated substantial international collaboration, particularly with researchers in the United States. Analysis of the 86 KNN-related publications revealed the rapid development of observational and topic-focused research areas within a relatively short period. Keyword clustering and temporal analyses demonstrated a progressive expansion of research interests as the database matured. Burst analysis identified emerging themes related to mortality and morbidity risk factors, neurodevelopmental outcomes, and predictive modeling using machine learning techniques. **Conclusions**: Bibliometric analysis demonstrated that research based on the KNN has largely followed global trends in neonatal research despite its relatively recent establishment. The KNN has successfully functioned as a nationwide research infrastructure facilitating collaborative knowledge generation and scientific productivity. Nevertheless, several underrepresented research areas and opportunities for broader international collaboration were identified. These findings may help guide future research strategies and maximize the impact of national neonatal registry-based healthcare systems.

## 1. Introduction

The Korean Neonatal Network (KNN), established in 2013, was created to systematically collect nationwide clinical data from very low birth weight infants (VLBWIs) admitted to neonatal intensive care units (NICUs) across South Korea, with the aim of improving neonatal care and outcomes. Initially comprising 60 NICUs and covering approximately 70% of VLBWIs born nationwide [1], the KNN has expanded to include 79 participating NICUs and data from more than 20,000 infants, representing over 80% of VLBWIs in South Korea [2].

The KNN database contains comprehensive clinical information, including perinatal characteristics, comorbidities, treatment practices, and long-term growth and developmental outcomes. Through continuous data collection and benchmarking, the network has supported quality improvement initiatives and evidence generation in neonatal care. Beyond its role as a clinical registry, the KNN has evolved into a research infrastructure that supports multicenter research, evidence generation, and data-driven healthcare decision-making.

Over the past decade, the KNN database has supported more than 200 publications spanning a broad range of neonatal topics. At the time of manuscript preparation, the KNN publication records listed 97 peer-reviewed publications. Following database retrieval and application of predefined eligibility criteria, including the exclusion of publications not indexed in the selected bibliographic databases and records that did not meet the study criteria, 86 publications were included in the present bibliometric analysis (Supplementary Table S1). These research outputs demonstrate how a nationwide clinical registry can transform routinely collected healthcare data into scientific evidence, facilitate multicenter collaboration, and support evidence-informed neonatal care. Evaluating these publication patterns may provide valuable insights into how large-scale healthcare data infrastructures support scientific productivity, collaborative knowledge generation, and the identification of future research priorities in neonatal medicine.

Bibliometrics is a field of research that uses quantitative analysis to describe publication patterns within a given field or body of literature [3]. Bibliometric methods are broadly categorized into evaluative and relational approaches. Relational bibliometrics has been widely applied in medical research to provide an overview of the relationships and networks among articles, authors, and institutions by analyzing the co-occurrence of bibliographic metadata. It helps researchers identify influential papers, key researchers, collaborative networks, and current research trends [4]. This approach has been widely applied in medical research, including studies on coronavirus disease 2019 (COVID-19) [5] and oncology research networks [6].

Bibliometric neonatological studies have focused on specific diseases and diagnostic procedures. For example, a bibliometric study on neonatal abstinence syndrome focused on research productivity and collaboration in the United States, Canada, and the United Kingdom [7]. Other studies in China have also conducted bibliometric analyses on various neonatal topics, such as retinopathy of prematurity [8], neonatal brain magnetic resonance imaging [9], and electroencephalograms [10]. To the best of our knowledge, this is the first bibliometric study to compare research output from a national neonatal registry (the KNN) with the global literature on VLBWIs. Unlike previous bibliometric studies that focused on individual neonatal diseases or specific clinical topics, the present study evaluated the scientific productivity and collaborative knowledge generation of a nationwide neonatal registry by directly comparing registry-based publications with the global neonatal literature. Accordingly, this study aimed to characterize research trends within the KNN and to evaluate how a nationwide neonatal registry has supported scientific productivity, collaborative knowledge generation, and future research priorities over the past decade. Because terminology describing this population varies across the literature, the search strategy incorporated standardized MeSH and Emtree terms together with commonly used synonyms to maximize the retrieval of relevant publications.

## 2. Materials and Methods

### 2.1. Data Collection

Data were collected from the Web of Science Core Collection database, covering literature published between 2015 and 2024 (search date: 5 June 2024). The Web of Science Core Collection was selected as the primary bibliographic database because it provides standardized citation metadata and comprehensive citation indexing, making it well suited for bibliometric analyses. To ensure objectivity and consistency of the study, the search strategy, study selection, and data preprocessing were independently reviewed by a medical librarian and two neonatology experts.

For the Global study, we searched the Author Keywords, Keywords Plus, and Title fields using the MeSH (on PubMed) terms (“Infant, Very Low Birth Weight” OR “Infant, Extremely Low Birth Weight” OR “Infant, Extremely Premature”) and Emtree (on Embase) terms (“very low birth weight” OR “extremely low birth weight” OR “extremely premature birth”), and their related synonyms. To comprehensively identify publications related to VLBWIs and extremely low birth weight infants (ELBWIs), additional search terms describing birth weight and gestational age were incorporated into the search strategy.

For the KNN study, publications were identified by searching the Title and Abstract fields using the single search term “Korean Neonatal Network”. Title and Abstract fields were selected because they provide the most consistent identification of studies explicitly based on the Korean Neonatal Network while minimizing retrieval of unrelated publications. The search was restricted to publications from 2015 to 2024, corresponding to the period since KNN data were first published, and to publication types classified as articles or reviews.

At the time of manuscript preparation, the KNN publication records contained 97 peer-reviewed publications. Among these, seven peer-reviewed articles published in journals not indexed in the bibliographic databases used in this study (Web of Science Core Collection, PubMed, and Scopus) were not retrievable through the predefined database search strategy. Consequently, 90 publications were identified through database searching. After restricting the publication type to articles and reviews, 89 publications remained. Manual screening was independently performed according to the predefined eligibility criteria. One publication that did not analyze KNN-derived data, one study conducted outside South Korea, and one conference abstract without a corresponding full-length peer-reviewed journal article were excluded, resulting in 86 KNN-related publications.

For both datasets, only articles and reviews were included, while animal studies were excluded. In addition, retracted publications were excluded from the Global dataset. Consequently, the final datasets comprised 7060 Global publications and 86 KNN publications. The complete search strategy and study selection process are presented in Supplementary Table S1.

For keyword analysis, the 86 eligible KNN publications were additionally searched in PubMed and Scopus to retrieve author keywords and indexed keywords. These databases were used to maximize keyword retrieval, improve keyword completeness, and reduce the possibility of missing indexed keywords because the number of KNN-related publications was relatively small, thereby enhancing the comprehensiveness of the keyword preprocessing procedure.

### 2.2. Keywords Preprocessing

In bibliographic information, keywords (author keywords, database-indexed keywords) may be represented differently in the text due to synonyms, spelling variants, abbreviations, and other factors. This, in turn, scatters the number of occurrences of keywords, affecting their weights and links with others; therefore, preprocessing is required to control the vocabulary.

Because terminology describing the target population varies across the literature, standardized MeSH and Emtree terms were used together with commonly used synonyms, including very low birth weight infants (VLBWIs), extremely low birth weight infants (ELBWIs), and extremely premature infants, to maximize the retrieval of relevant publications. Artificial intelligence- and machine learning-related terms included “artificial intelligence”, “machine learning”, “artificial neural network”, “deep neural network”, and “support vector machine”. These standardized definitions formed the basis of the subsequent keyword preprocessing procedure. The keywords were processed in three detailed steps: (1) text editing, correcting typos and indexing errors, unifying singular and plural terms into their most used forms, expanding abbreviations, fixing inverted terms, and truncating MeSH subheadings; (2) merging and consolidating synonyms of medically equivalent concepts into a single representative term; and (3) selection, excluding terms that are too non-specific to represent the research topic. The text was edited using OpenRefine (ver. 3.8.0) as the main tool. The merging process was initially organized using OpenRefine and then directly clustered into semantically identical terms. The criteria for selecting a representative term to group a concept were based on clinical judgment, followed by keywords with the highest number of occurrences, which are commonly used in this specialty. To improve reproducibility, all preprocessing decisions were independently reviewed by a medical librarian and two neonatology experts. Disagreements were resolved through discussion and consensus using standardized controlled vocabularies (MeSH and Emtree). Ambiguous keywords were organized into controlled vocabulary by referring to the hierarchical structure of the medical thesaurus (MeSH, Emtree) as a consistent criterion. The selection of keywords was performed by establishing the following criteria, reviewing keywords that met the predefined occurrence thresholds (Global top 1000, all KNN research keywords), and then excluding nonspecific terms through discussion and consensus between the authors. The exclusion criteria were as follows: (1) terms that expressed the topic of the article too broadly, such as death, disease, and disorder; (2) terms that referred to the field of pediatric and premature infant research and the topic itself, such as neonate, newborn, and premature; (3) terms related to the type of publication, such as articles, case reports, and reviews; and (4) terms that were scientific concepts that were too general, such as age, life, and rate. However, we did not exclude terms that were not specific, such as risk factors, mortality, or incidence, because we believed that they could provide information about the outcome of the study if they were relevant to the study’s interest. Representative examples of synonym grouping, abbreviation normalization, and excluded generic terms used during keyword preprocessing are provided in Supplementary Table S2.

### 2.3. Data Analysis and Visualization

Bibliometric relationships were analyzed and visualized using publicly available software.

We analyzed and visualized the co-authorship by countries of the Global data using VOSviewer (ver. 1.6.20), software that constructs and visualizes bibliometric networks. In this analysis, the counting method was fractional counting, and documents co-authored by several countries were ignored (maximum per document: default 25) to reduce the impact of articles co-authored by multiple authors on the network [11]. The other setting was the minimum number of documents per country: 3.

Keyword co-occurrence analysis was performed using VOSviewer (ver. 1.6.20). Keywords occurring at least three times after preprocessing were included in the network analysis for both datasets. This threshold was selected to reduce noise while retaining sufficient keywords to characterize the overall research landscape. These descriptions are provided to facilitate interpretation of the bibliometric network visualizations by readers unfamiliar with bibliometric software. In the network visualizations, each node represents a keyword, with node size proportional to its frequency of occurrence. Links between nodes indicate co-occurrence relationships, while the distance between nodes reflects the strength of their association, with more closely positioned nodes indicating stronger relationships. Nodes with similar co-occurrence patterns are grouped into clusters representing related research themes. The analysis was presented as a “Network” visualization, which shows clustering based on the number of co-occurrences between keywords, and an “Overlay” visualization, which shows the emergence of research topics over time by scoring the average year of appearance of keywords. In addition, we exported the map data containing the occurrences of each keyword, average publication year, and cluster information from the above KNN study analysis and used the data to generate a timeline visualization integrating the bibliometric results using the visualization tool Scimago Graphica (ver. 1.0.43).

The burst analysis was performed using CiteSpace (ver. 6.3.R1) to identify keywords that showed statistically significant frequency spikes over short time intervals [12] in both Global and KNN studies for the entire period of 2015–2024, indicating a sharp increase in interest in the field [13] of VLBWIs research. Burst analysis is based on Kleinberg’s burst detection algorithm [14]; the parameter γ among various configuration values ranges from 0 to 1, with values closer to 0 emphasizing burst events occurring within shorter time intervals [15]. The parameter was set to γ = 0.3 to identify a sufficient number of burst terms while considering the relatively small KNN dataset and the 10-year study period. A total of 17 burst terms were identified, and the same γ value and number of burst terms were applied to the Global dataset to ensure methodological consistency. For the Global study, the inclusion criteria were set with a scale factor k = 10 for the g-index, which allows for the inclusion of as many nodes as possible in this software version; for both studies, term sources included Author Keywords and Keywords Plus as preprocessed data. Additionally, we used the same software to plot the timeline of clusters based on the year of the first appearance of keywords. The comparison between the Global and KNN datasets was intended as an exploratory, descriptive bibliometric analysis to identify similarities and differences in research trends rather than to evaluate research performance or establish a ranking between the two datasets.

This study did not involve human subjects; therefore, approval from the Institutional Review Board was not required.

## 3. Results

### 3.1. Country Distribution of Documents

**Figure 1:**
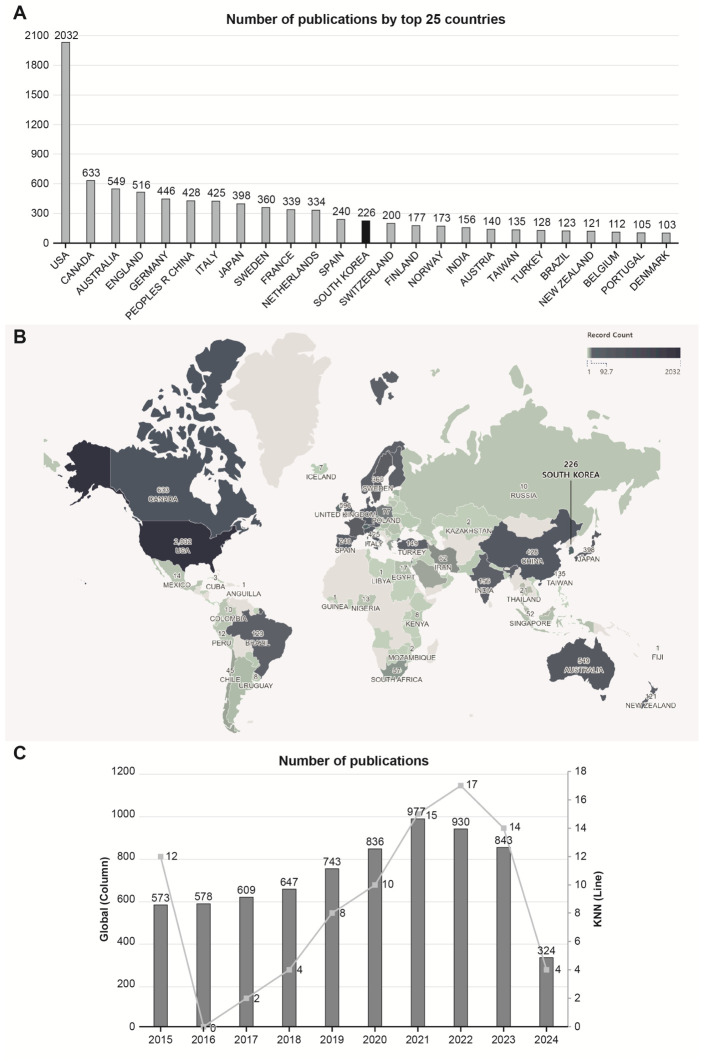
Number of publications by country in the Global study. (**A**) Bar graph, (**B**) world map, (**C**) proportion of KNN publications within the Global dataset.

**Figure 2:**
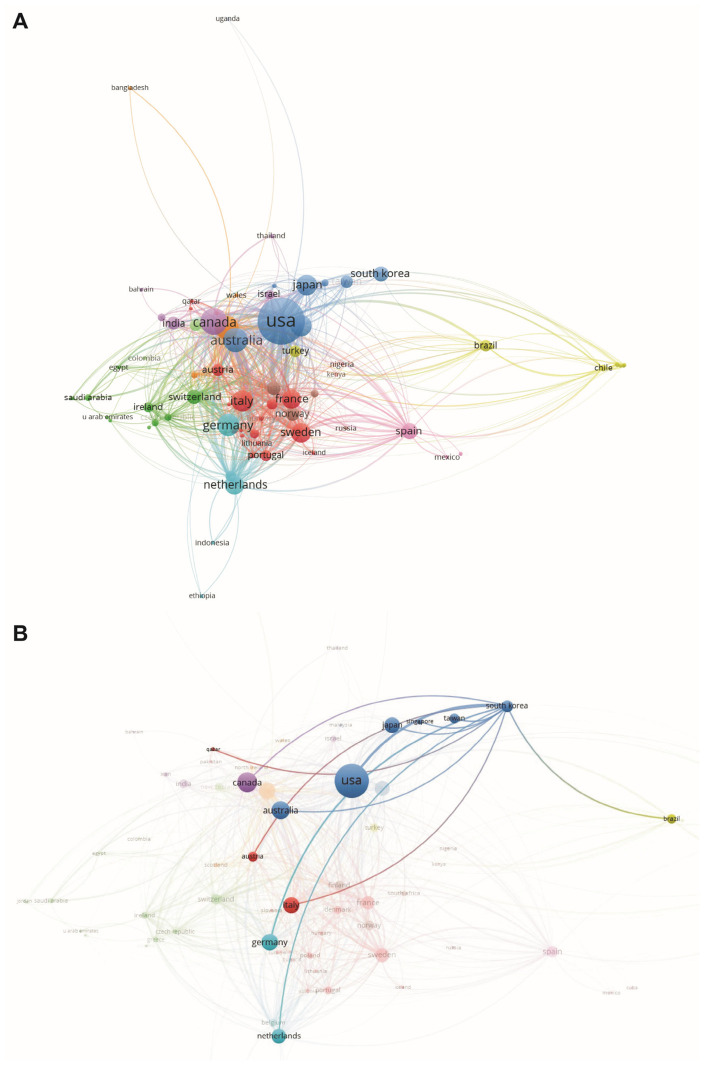
Visualization of the international co-authorship network (73 items, 12 clusters, 810 links, total link strength = 2014.00). Node size is proportional to the number of publications contributed by each country, link thickness represents the strength of international collaboration, and clusters represent groups of closely collaborating countries. (**A**) All countries, (**B**) South Korea and its international collaborations (226 publications, 12 links, total link strength = 10.00).

Among the 7060 publications that met the inclusion criteria using the keywords VLBWIs and ELBWIs, as expected, the United States was the leading country with the most publications meeting the inclusion criteria (2032 publications). Canada (633), Australia (549), and South Korea ranked 13th with 226 publications (Figure 1). An analysis of co-authorship networks by country revealed extensive collaboration between authors from the United States, England, and Canada. Twelve countries were identified within South Korea (Figure 2).

### 3.2. Analysis Using Top Keywords with the Strongest Citation Bursts

**Figure 3:**
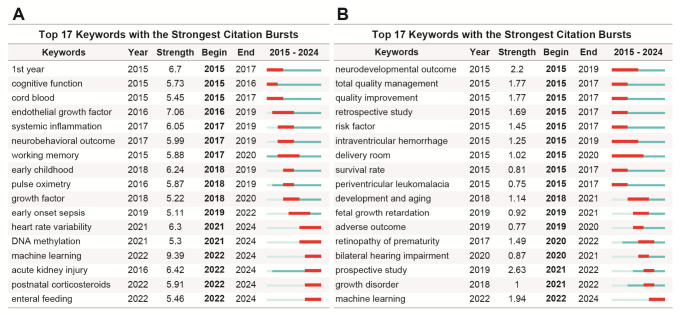
Burst analysis comparison of Global and KNN studies. (**A**) Global and (**B**) KNN studies.

When analyzing global published studies (γ = 0.3, based on Top 17), keywords related to research on specific treatments such as cord blood (2015) and endothelial growth factor (2016) were found. Additionally, keywords associated with development, such as cognitive function (2015), neurobehavioral outcome (2017), working memory (2017), and keywords related to infection, including systemic inflammation (2017) and early onset sepsis (2019), gradually emerged. Recently, burst analysis revealed keywords related to various topics for each organ, such as enteral feeding (2022) and acute kidney injury (2022), along with keywords related to prediction, such as machine learning (2022). Keywords related to treatment, including cord blood (2015), endothelial growth factor (2016), growth factor (2018), and postnatal corticosteroids (2022), continued to appear (Figure 3A).

When the target was limited to the KNN database, keywords related to various topics, including neurodevelopmental outcome (2015), risk factor (2015), and primary outcomes such as intraventricular hemorrhage (2015), survival rate (2015), and periventricular leukomalacia (2015), appeared simultaneously. Subsequently, neurodevelopment-related keywords emerged, including development and aging (2018), adverse outcome (2019), retinopathy of prematurity (2020), bilateral hearing impairment (2020), and growth disorder (2021). Additionally, the keyword machine learning (2022) appeared, similar to the global analysis (Figure 3B).

### 3.3. Analysis Using Term Maps on the KNN: Network Visualization and Overlaying Analysis

#### 3.3.1. Network Visualization

**Figure 4:**
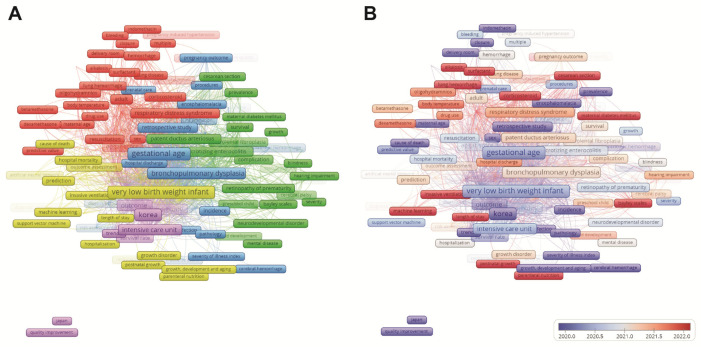
Keywords co-occurrence analysis of the KNN dataset (128 items, 5 clusters, 4177 Links, and a total link strength of 11,496). Node size is proportional to keyword occurrence, node color indicates the average publication year, and clusters represent groups of closely related research topics. (**A**) Network visualization (clustering). (**B**) Overlay visualization (average publication year of keywords).

The clusters represent the following research areas in order of frequency of occurrence (Figure 4A):

1. Red cluster represents perinatal factors of VLBWI with terms such as: “apgar score,” “respiratory distress syndrome,” “chorioamnionitis,” “maternal hypertension,” “resuscitation,” “corticosteroid,” “oligohydramnios,” “surfactant,” and “premature rupture of fetal membrane.”
2. Green cluster represents morbidity of VLBWI with terms such as: “necrotizing enterocolitis,” “brain hemorrhage,” “patent ductus arteriosus,” “retinopathy of prematurity,” “complication,” “survival,” “cerebral palsy,” “bayley scales,” “prevalence,” “pulmonary hypertension,” and “neurodevelopmental disorder.”
3. Blue cluster represents neurodevelopmental outcomes of VLBWI with terms such as: “gestational age,” “bronchopulmonary dysplasia,” “risk factor,” “sepsis,” “incidence,” “disease severity,” “encephalomalacia,” “neurodevelopmental outcome,” “periventricular leukomalacia,” “intraventricular hemorrhage,” and “hospital discharge.”
4. Yellow cluster represents mortality with artificial intelligence of VLBWI with terms such as: “very low birth weight infant,” “prospective study,” “small for gestational age,” “prediction,” “machine learning,” “hospital mortality,” “invasive ventilation,” “clinical outcome,” “growth disorder,” “outcome assessment,” “length of stay,” “spontaneous intestinal perforation,” “air leak syndrome,” “cause of death,” and “growth, development and aging.”
5. Purple cluster represents general trends analysis of VLBWI with terms such as: “korea,” “mortality,” “outcome,” “morbidity,” “intensive care unit,” “extremely low birth weight infant,” “trends,” “survival rate,” “epidemiology,” “health care quality,” “japan,” “organization and management,” and “quality improvement.”

#### 3.3.2. Overlay Visualization

Overlay visualization demonstrated chronological changes in keyword occurrence across the study period. Earlier publications were characterized by keywords related to mortality, major neonatal morbidities, and associated risk factors, whereas more recent publications more frequently included keywords related to long-term neurodevelopmental outcomes, nutrition, and treatment strategies. More recent publications showed the emergence of artificial intelligence- and machine learning-related keywords, which were identified in 10 of the 86 KNN publications (Figure 4). Thematic distributions of keywords in the Global and KNN datasets are quantitatively summarized in Supplementary Table S3.

### 3.4. Timeline Map of Keyworks

**Figure 5:**
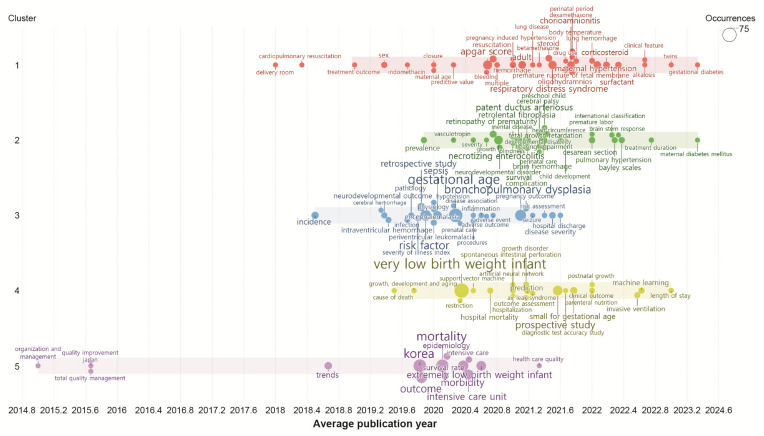
Timeline visualization of keywords by average publication year in the KNN study. Node size, links, and cluster definitions are as described in Figure 4.

When analyzing keywords that appeared simultaneously by period, focusing on high-frequency keywords, terms corresponding to each field began to occur simultaneously from 2018, four years after the establishment of KNN (cluster 1 [Red—perinatal factors], cluster 2 [Green—morbidity], cluster 3 [Blue—neurodevelopmental outcomes], cluster 4 [Yellow—mortality]). The high-frequency keywords that occurred over time included those related to general trend analysis and neurodevelopmental outcomes, followed by those associated with mortality and morbidity. Keywords related to the overall objectives of the KNN, including epidemiology, healthcare quality, and trends (cluster 5 [Purple—general trends analysis]) appeared throughout the study period (Figure 5). These findings should be interpreted cautiously because underrepresented topics may partly reflect the evolving maturity of the registry and the limited number of eligible publications included in the present study, rather than a lack of research interest.

## 4. Discussion

This study used bibliometric network analysis to evaluate how the Korean Neonatal Network (KNN), a nationwide neonatal healthcare registry, has transformed routinely collected clinical data into scientific output and collaborative knowledge generation over the past decade. By comparing KNN publications with the global literature regarding on VLBWIs, we characterized research trends, international collaboration, thematic evolution, and emerging research topics through co-authorship and keyword network analyses.

Globally, South Korea ranked 13th in publication output in VLBWI research, with the strongest collaborative links identified with North American countries. These collaborations may reflect longstanding academic partnerships and the increasing international visibility of KNN-based neonatal research. The expanding collaboration network also suggests that the KNN has matured into a national research infrastructure supporting multicenter research. Because multinational collaboration enhances statistical power, improves external validity and the generalizability of research findings, and enables comparisons across different healthcare systems [16], further international registry collaboration may strengthen future neonatal research. In particular, integrating nationwide neonatal registries may be especially valuable for studies requiring large-scale longitudinal datasets, including long-term neurodevelopmental outcomes, predictive modeling using machine learning, comparative effectiveness research, and relatively uncommon neonatal conditions.

Keyword co-occurrence and overlay analyses demonstrated that KNN-based research has encompassed perinatal-maternal factors, neonatal morbidity, neurodevelopmental outcomes, mortality, and general trends. As the KNN database has matured, keywords related to traditional observational research (morbidity, mortality, and associated risk factors) and long-term neurodevelopmental outcomes emerged concurrently, reflecting the broadening scope of registry-based research. Compared with the Global dataset, some research themes appeared less frequently in the KNN literature. However, these findings should be interpreted cautiously because the global literature comprises studies conducted across diverse healthcare systems, funding structures, registry infrastructures, and publication practices, all of which may influence research priorities and publication patterns. Therefore, the comparison was intended to identify broad similarities and differences in research trends rather than to compare research performance or healthcare systems directly. The Global dataset contained a wider range of keywords related to ELBWIs, with more distinct thematic clusters involving perinatal-maternal factors, neurodevelopment, nutrition, bronchopulmonary dysplasia, infection, and treatment-related interventions (Figure S1A). By comparison, several emerging research areas—including precision medicine, advanced predictive modeling, nutrition-related interventions, and international collaborative research—were less frequently represented in KNN publications. This pattern is likely explained by the relatively recent establishment and evolving maturity of the KNN, together with the limited number of eligible publications included in the present analysis, rather than by a lack of research interest. As additional longitudinal data continue to accumulate, broader research themes are expected to emerge, further enhancing the scientific value and international impact of the registry. Another notable finding was the recent emergence of artificial intelligence- and machine learning-related keywords. Although these terms were identified in only 10 of the 86 KNN publications, they represent an emerging research direction rather than an established research focus. As nationwide neonatal registries continue to accumulate high-quality longitudinal clinical data, artificial intelligence and machine learning approaches may support the future development and evaluation of predictive models that could ultimately inform clinical decision-making.

This was consistent with the keyword appearance period confirmed in the burst analysis. Compared with the global analysis (Figure S2A) of the keyword timeline additionally analyzed using CiteSpace, the appearance period of topic-related keywords in the KNN study was clearly delineated (Figure S2B). Cheung [17] and Carra et al. [18] reported that trends in big data analysis initially evolved from traditional observational studies to changes in treatment, assessment of safety and efficacy of modified treatment, precision medicine, development of predictive models, and personalized treatment. In the global analysis, many keywords related to growth and development appeared as cold-colored keywords, whereas recent studies, represented as warm-colored keywords, showed a prevalence of keywords related to the development of predictive models using artificial intelligence. A similar trend appears to be emerging in neonatal research, including studies involving VLBWIs (Figure S1B). These findings suggest that future KNN-based research may continue to follow similar trends. The timeline visualization organized KNN keywords according to their average year of appearance with each co-occurrence cluster, enabling visualization of temporal evolution of research topics. This approach allows for a macroscopic understanding of the overall changes in research topics, while also providing the strength of examining detailed aspects through the nodes within each individual cluster. Global research was analyzed for comparison with KNN research and to understand trends. The results are presented in a supplementary format.

This study was conducted using a relatively small number of KNN publications accumulated over a relatively short period and focused on a specific neonatal registry. To address these limitations, we implemented a comprehensive search strategy (1) establishing a multi-faceted keyword search strategy (including birth weight and gestational age terms to identify relevant publications on extremely preterm infants for the Global study) and (2) retrieving differently indexed keywords from multiple databases (PubMed and Scopus) to maximize keyword completeness and diversify topic identification for the KNN study. This approach is consistent with previous bibliometric studies in medicine, which have suggested that comprehensive search strategies across multiple databases, similar to those used in systematic reviews, can improve the robustness of bibliometric analysis [19].

Nevertheless, several limitations should be acknowledged. First, although the Web of Science Core Collection was selected as the primary bibliographic database because of its standardized citation metadata and compatibility with bibliometric software, relevant publications not indexed in the selected databases may have been missed. Second, KNN-related publications were identified using the Title and Abstract fields, which may have resulted in the under-identification of studies that utilized KNN-derived data without explicitly mentioning the KNN. Third, keyword preprocessing involved manual synonym merging, abbreviation normalization, and exclusion of generic terms based on expert consensus, which may have introduced a degree of subjectivity. To improve consistency and reproducibility, all preprocessing decisions were independently reviewed by a multidisciplinary team using predefined criteria and standardized controlled vocabularies (MeSH and Emtree). Although formal inter-rater reproducibility testing was not performed, these procedures were intended to enhance the transparency and consistency of the keyword preprocessing process. Finally, bibliometric analyses evaluate publication patterns, collaboration networks, and research trends, rather than the methodological quality of individual studies or their direct impact on clinical outcomes. Therefore, comparisons between the Global and KNN datasets should be interpreted as differences in research characteristics and scientific productivity rather than direct measures of healthcare quality or clinical effectiveness, particularly given the differences in healthcare systems, research environments, and publication practices across countries.

The increasing number and diversity of KNN-based publications demonstrate the scientific productivity of the nationwide neonatal registry. The KNN has evolved beyond a clinical registry into a sustainable research platform that facilitates multicenter collaboration and supports knowledge generation in neonatal medicine. By identifying emerging research priorities and promoting collaborative knowledge generation, registry-based networks such as the KNN provide valuable evidence for neonatal research. Although these findings suggest that registry-based research may provide evidence to support healthcare quality improvement and evidence-informed policy development, the present bibliometric analysis evaluated publication patterns rather than healthcare outcomes. Therefore, the direct impact of KNN research on clinical practice or patient outcomes cannot be determined from the present study. Our findings provide insight into how national clinical registries support scientific productivity, multicenter collaboration, and knowledge dissemination, while generating evidence that may inform future healthcare quality improvement initiatives.

To the best of our knowledge, this is the first bibliometric study to compare research output from the KNN with global publications in neonatal medicine. Future studies integrating multiple bibliographic databases, expanding international neonatal registry collaborations, and incorporating clinical outcome data may further improve the completeness, generalizability, and clinical relevance of bibliometric analyses in this field.

## 5. Conclusions

Bibliometric analysis demonstrated that the Korean Neonatal Network has evolved beyond a national clinical registry to become a productive healthcare research infrastructure supporting scientific output, multicenter collaboration, and knowledge generation in neonatology. Although KNN-based research has largely followed global research trends, several underrepresented areas and opportunities for broader international collaboration remain. These findings provide insight into how national healthcare data platforms support scientific productivity and knowledge generation that may ultimately inform healthcare quality improvement and evidence-informed policy development. The bibliometric framework presented in this study may also be applied to other national clinical registries to evaluate research productivity, collaboration networks, thematic evolution, and emerging research priorities. Such comparative bibliometric evaluations may help optimize the scientific value of nationwide healthcare databases and support evidence-informed research planning across different healthcare systems.

## Data Availability

Data are contained within the article or Supplementary Materials. The datasets generated and/or analyzed during the current study are not publicly available but are available from the corresponding author upon reasonable request and subject to review of the request.

## Supplementary Materials

The following supporting information can be downloaded at: https://www.mdpi.com/article/10.3390/healthcare14142188/s1, Figure S1. Keyword co-occurrence analysis of the Global study. (A) Network visualization showing clusters of keywords based on co-occurrence relationships. Each node represents a keyword, and the size of the node is proportional to its frequency of occurrence. Links between nodes indicate co-occurrence relationships, and different colors represent distinct research clusters. (B) Overlay visualization illustrating the temporal evolution of keywords. The color of each node represents the average publication year, with blue indicating earlier studies and yellow indicating more recent studies. Figure S2. Timeline and burst analysis of keywords in Global and KNN studies. (A) Timeline visualization of keyword clusters in the Global study, showing the temporal distribution and duration of major research topics. (B) Timeline visualization of keyword clusters in the KNN study, demonstrating more distinct and sequential emergence of research topics compared to the Global study. Red bars indicate periods of significant increases in keyword frequency, reflecting emerging research trends. Table S1. Search strategies and Study selection process for Global and KNN studies of Web of Science. Table S2. Representative examples of keyword preprocessing, synonym grouping, abbreviation normalization, and excluded generic terms used during bibliometric data preprocessing. Table S3. Quantitative comparison of the distribution of major thematic domains between the Global and KNN datasets. Table S4. Interactive bibliometric network visualizations.

## Abbreviations

The following abbreviations are used in this manuscript:

KNN	Korean Neonatal Network
VLBWI	Very Low Birth Weight Infant
ELBWI	Extremely Low Birth Weight Infant
NICU	Neonatal Intensive Care Unit
WoS	Web of Science
