# Supplementary material for: Evaluating a Nationwide Neonatal Research Infrastructure: A Bibliometric Network Analysis of the Korean Neonatal Network
Source: Healthcare (Basel). 2026 Jul 20;14(14):2188. doi: 10.3390/healthcare14142188 (PMC13410123; doi:10.3390/healthcare14142188)
Supplement: Supplementary file 1 [file healthcare-14-02188-s001.zip › healthcare-4415568-supplementary.pdf]

Supplementary Material

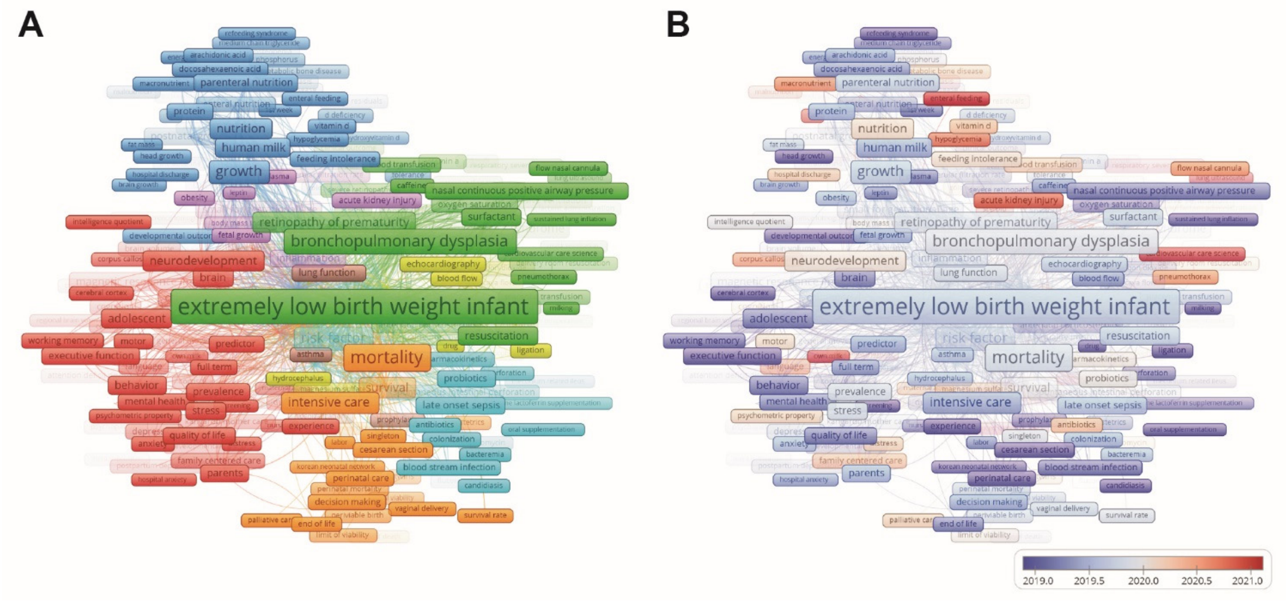

Figure S1. Keyword co-occurrence analysis of the Global study. (A) Network visualization showing clusters of keywords based on co-occurrence relationships. Each node represents a keyword, and the size of the node is proportional to its frequency of occurrence. Links between nodes indicate co-occurrence relationships, and different colors represent distinct research clusters. (B) Overlay visualization illustrating the temporal evolution of keywords. The color of each node represents the average publication year, with blue indicating earlier studies and yellow indicating more recent studies.

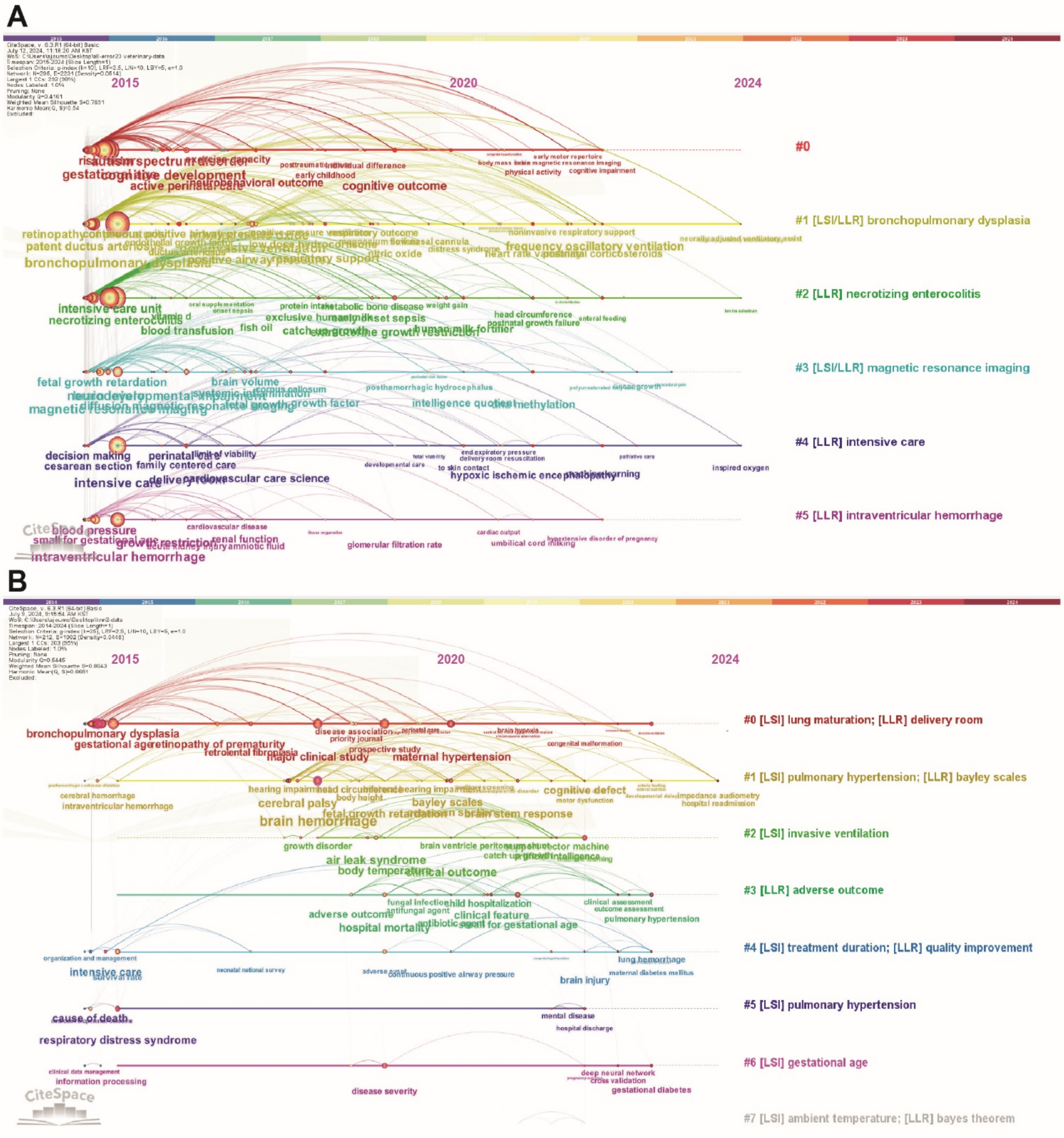

Figure S2. Timeline and burst analysis of keywords in Global and KNN studies. (A) Timeline visualization of keyword clusters in the Global study, showing the temporal distribution and duration of major research topics. (B) Timeline visualization of keyword clusters in the KNN study, demonstrating more distinct and sequential emergence of research topics compared to the Global study. Red bars indicate periods of significant increases in keyword frequency, reflecting emerging research trends.

Table S1. Search strategies and Study selection process for Global and KNN studies of Web of Science.

**Search Date 2024-06-05**		
No.	Query	Results
**Korean Neonatal Network (KNN)**		
#1	(TI="korean neonatal network*" OR AB="korean neonatal network*")	90
#2	DT=(Article) OR DT=(Review)	20,841,487
#3	#1 AND #2	89
	Exclude (-3)<br>- Not using KNN data<br>- Not studied in South Korea<br>- Congress abstract	**86**
**Gobal**		
#1	AK=("Infant, Very Low Birth Weight" OR "Infant, Extremely Low Birth Weight" OR "Infant, Extremely Premature" OR "very low birth weight" OR "extremely low birth weight" OR "extremely premature birth") OR KP=("Infant, Very Low Birth Weight" OR "Infant, Extremely Low Birth Weight" OR "Infant, Extremely Premature" OR "very low birth weight" OR "extremely low birth weight" OR "extremely premature birth")	1425
#2	TI=("very low birth weight*" OR "very low birthweight*" OR "vlbw*" OR "extremely low birth weight*" OR "extremely low birthweight*" OR "elbw*")	2905
#3	TI=(("1.5 kg" OR "1.5kg" OR "1.5 kilograms" OR "1500 g" OR "1500g" OR "1500 grams" OR "1,500 g" OR "1,500g" OR "1,500 grams" OR "1250 g" OR "1250g" OR "1250 grams" OR "1,250 g" OR "1,250g" OR "1,250 grams" OR "1 kg" OR "1kg" OR "1 kilograms" OR "1000 g" OR "1000g" OR "1000 grams" OR "1,000 g" OR "1,000g" OR "1,000 grams" OR "3 pounds" OR "4 ounces" OR "4 oz" OR "4oz" OR "3 ounces" OR "3 oz" OR "3oz" OR "2 lb" OR "2lb") NEAR/2 ("neonat*" OR "infant*" OR "born" OR "newborn*" OR "new born*"))	83
#4	(TI=(("29 week*" OR "28 week*" OR "< 29" OR "< 28" OR "<29" OR "<28") NEAR/2 ("neonat*" OR "infant*" OR "born" OR "newborn*" OR "new born*")) AND TI=("week*")) NOT TI=("29 day*" OR "28 day*")	168
#5	TI=(("1.5 kg" OR "1.5kg" OR "1.5 kilograms" OR "1500 g" OR "1500g" OR "1500 grams" OR "1,500 g" OR "1,500g" OR "1,500 grams" OR "1250 g" OR "1250g" OR "1250 grams" OR "1,250 g" OR "1,250g" OR "1,250 grams" OR "1 kg" OR "1kg" OR "1 kilograms" OR "1000 g" OR "1000g" OR "1000 grams" OR "1,000 g" OR "1,000g" OR "1,000 grams" OR "3 pounds" OR "4 ounces" OR "4 oz" OR "4oz" OR "3 ounces" OR "3 oz" OR "3oz" OR "2 lb" OR "2lb") NEAR/5 ("weight*" OR "birthweight*") NEAR/5 ("neonat*" OR "infant*" OR "born" OR "newborn*" OR "new born*" OR "preterm*" OR "premature"))	55
#6	TI=(("extreme*" OR "very") NEAR/2 ("preterm*" OR "premature" OR "infant*"))	4596
#7	AK=("very low birth weight*" OR "very low birthweight*" OR "vlbw*" OR "extremely low birth weight*" OR "extremely low birthweight*" OR "elbw*") OR KP=("very low birth weight*" OR "very low birthweight*" OR "vlbw*" OR "extremely low birth weight*" OR "extremely low birthweight*" OR "elbw*")	1888
#8	AK=(("1.5 kg" OR "1.5kg" OR "1.5 kilograms" OR "1500 g" OR "1500g" OR "1500 grams" OR "1,500 g" OR "1,500g" OR "1,500 grams" OR "1250 g" OR "1250g" OR "1250 grams" OR "1,250 g" OR "1,250g" OR "1,250 grams" OR "1 kg" OR "1kg" OR "1 kilograms" OR "1000 g" OR "1000g" OR "1000 grams" OR "1,000 g" OR "1,000g" OR "1,000 grams" OR "3 pounds" OR "4 ounces" OR "4 oz" OR "4oz" OR "3 ounces" OR "3 oz" OR "3oz" OR "2 lb" OR "2lb") NEAR/2 ("neonat*" OR "infant*" OR "born" OR "newborn*" OR "new born*")) OR KP=(("1.5 kg" OR "1.5kg" OR "1.5 kilograms" OR "1500 g" OR "1500g" OR "1500 grams" OR "1,500 g" OR "1,500g" OR "1,500 grams" OR "1250 g" OR "1250g" OR "1250 grams" OR "1,250 g" OR "1,250g" OR "1,250 grams" OR "1 kg" OR "1kg" OR "1 kilograms" OR	0

	"1000 g" OR "1000g" OR "1000 grams" OR "1,000 g" OR "1,000g" OR "1,000 grams" OR "3 pounds" OR "4 ounces" OR "4 oz" OR "4oz" OR "3 ounces" OR "3 oz" OR "3oz" OR "2 lb" OR "2lb") NEAR/2 ("neonat*" OR "infant*" OR "born" OR "newborn*" OR "new born*"))	
#9	((AK=(("29 week*" OR "28 week*" OR "< 29" OR "< 28" OR "<29" OR "<28") NEAR/2 ("neonat*" OR "infant*" OR "born" OR "newborn*" OR "new born*")) AND AK=("week*")) NOT AK=("29 day*" OR "28 day*")) OR ((KP=(("29 week*" OR "28 week*" OR "< 29" OR "< 28" OR "<29" OR "<28") NEAR/2 ("neonat*" OR "infant*" OR "born" OR "newborn*" OR "new born*")) AND KP=("week*")) NOT KP=("29 day*" OR "28 day*"))	1
#10	AK=(("extreme*" OR "very") NEAR/2 ("preterm*" OR "premature" OR "infant*")) OR KP=(("extreme*" OR "very") NEAR/2 ("preterm*" OR "premature" OR "infant*"))	2016
#11	#1 OR #2 OR #3 OR #4 OR #5 OR #6 OR #7 OR #8 OR #9 OR #10	8773
#12	PY=(2015-2024)	29,254,496
#13	DT=(Article) OR DT=(Review)	20,841,487
#14	#11 AND #12 AND #13	7,067
	Exclude (-7)<br>- Researches in agriculture, dairy, and animal sciences (-4)<br>- Retracted article and reviews (-3)	**7060**

Table S2. Representative examples of keyword preprocessing, synonym grouping, abbreviation normalization, and excluded generic terms used during bibliometric data preprocessing.

**Representative synonym groups**		
Original keywords	Representative term	Action
Infant, Very Low Birth Weight; very low birth weight; very low birth weight; very low birthweight; vlbw	VLBWI	Synonym merged
1.5 kg; 1.5kg; 1.5 kilograms; 1500 g; 1500g; 1500 grams; 1500 g; 1500g; 1500 grams	1500 g	Synonym merged
Infant, Extremely Low Birth Weight; Infants, Extremely Premature; extremely low birth weight; extremely low birthweight; extremely low birth weight; extremely premature birth; elbw	ELBWI	Synonym merged
1 kg; 1kg; 1 kilograms; 1000 g; 1000g; 1000 grams; 1000 g; 1000g; 1000 grams	1000 g	Synonym merged
weight; birthweight	Birth weight	Synonym merged
Neonate; infant; born; newborn; new born	neonate	Synonym merged
Preterm; premature	preterm	Synonym merged
NEC; necrotizing enterocolitis	Necrotizing enterocolitis	Synonym merged
RDS; respiratory distress syndrome	Respiratory distress syndrome	Synonym merged
PDA; patent ductus arteriosus	Patent ductus arteriosus	Synonym merged
BPD; bronchopulmonary dysplasia	Bronchopulmonary dysplasia	Synonym merged

**Excluded generic terms**	
Excluded generic terms	Reason
Study	Generic methodological term
Outcome	Generic outcome descriptor
Infant	Generic population descriptor
Child	Generic population descriptor
Patient	Generic population descriptor
Analysis	Generic methodological term
Data	Generic methodological term

**Abbreviation normalization**	
Original	Standardized
NICU	Neonatal intensive care unit
MRI	Magnetic resonance imaging
EEG	Electroencephalography
VLBWI	Very low birth weight infant
ELBWI	Extremely low birth weight infant

Only representative examples are shown; the complete keyword preprocessing procedure was applied consistently across all retrieved records

Table S3. Quantitative comparison of the distribution of major thematic domains between the Global and KNN datasets.

Thematic domain	Global		KNN	
	Keywords occurrences Total n = 13,669	Number of articles Total n = 7060	Keywords occurrences Total n = 975	Number of articles Total n = 86
Perinatal factors	652 (4.8%)	555 (7.9%)	124 (12.7%)	44 (51.2%)
Morbidity	2052 (15.0%)	1851 (26.2%)	132 (13.5%)	56 (65.1%)
Neurodevelopment	2961 (21.7%)	2387 (33.8%)	228 (23.4%)	77 (90.6%)
Mortality and predictive analytics	2129 (15.6%)	1992 (28.2%)	190 (19.5%)	79 (91.9%)
General trends (included registry-related, epidemiological, and healthcare system-related keywords)	5875 (43.0%)	3815 (54.0%)	301 (30.9%)	80 (93.0%)

Keywords were classified into predefined thematic domains according to the keyword preprocessing procedure. For each thematic domain, both the total number of keyword occurrences and the number of unique publications were calculated separately for the Global and KNN datasets. Publications containing multiple keywords within the same thematic domain were counted only once for that domain. Percentages for keyword occurrences were calculated relative to the total number of keyword occurrences across all thematic domains within each dataset. Percentages for publications were calculated relative to the total number of publications included in each dataset (Global, n = 7060; KNN, n = 86).

Table S4. Interactive bibliometric network visualizations.

Figure	The map available online
2A	https://tinyurl.com/296lkjbe
2B	https://tinyurl.com/2ccmnblv
4A	https://tinyurl.com/25ws2kol
4B	https://tinyurl.com/2d69ax8m.
